# Understanding the Traumatic Impact of Serious Chronic Illness

**DOI:** 10.1192/j.eurpsy.2024.767

**Published:** 2024-08-27

**Authors:** M. Theodoratou, H. Diamanti

**Affiliations:** ^1^Social Sciences, Hellenic Open University, Patras, Greece

## Abstract

**Introduction:**

The diagnosis of a severe chronic illness represents a deeply impactful traumatic event, frequently giving rise to initial adverse consequences that can manifest as post-traumatic stress. The duration and characteristics of these effects exhibit considerable variation among individuals.

**Objectives:**

This study aims to explore the levels of post-traumatic stress, post-traumatic growth, and psychosocial adaptation among individuals coping with chronic diseases.

**Methods:**

This cross-sectional study involved 92 participants with chronic illnesses, recruited through convenience and snowball sampling. Data collection utilized an online questionnaire that included both demographic questions to provide a comprehensive understanding of participants’ experiences, as well as psychometric scales for measuring post-traumatic stress, post-traumatic growth, and psychosocial adaptation.

Instruments used :

1.PTSD Checklist for DSM-5 (PCL-5)

2.Posttraumatic Growth Inventory (PTGI) and Tedeschi and Calhoun Posttraumatic Growth Inventory (TCGI).

3.Psychosocial Adjustment to Illness Scale (PAIS).

Analysis included descriptive statistics and inductive analysis using SPSS (p < 0.05). Ethical considerations were observed, with informed consent and data confidentiality.

**Results:**

The study revealed the presence of low to moderate levels of post-traumatic stress (M= 2.45), moderate levels of post-traumatic growth (M= 2.90), and moderate levels of psychosocial adaptation in various aspects of participants’ lives, including work (M= 2.36), sexuality (M= 2.11), sociability (M= 2.28), relationships with partners and family members (M= 1.92), and perception of their health (M= 1.94). Furthermore, the overall psychosocial situation of the participants was found to range from low to moderate (M= 2.48). Notably, individuals with fewer chronic illnesses tended to experience lower levels of post-traumatic stress and exhibited less adaptation in their work. Additionally, higher levels of post-traumatic growth were observed in women and patients with higher educational backgrounds. The analysis revealed a positive and statistically significant correlation (sig.<0.05) between post-traumatic stress, post-traumatic growth, and various dimensions of psychosocial adjustment among the participants.

**Conclusions:**

A chronic illness diagnosis can be deeply traumatic, potentially causing post-traumatic stress. However, it’s crucial to understand that this doesn’t diminish the possibility of post-traumatic growth and effective psychosocial adaptation. To foster this positive path, individuals must receive holistic psychological and emotional support, along with essential social assistance as they navigate life with chronic diseases.

**Disclosure of Interest:**

None Declared

